# Repeated mild traumatic brain injury causes sex-specific increases in cell proliferation and inflammation in juvenile rats

**DOI:** 10.1186/s12974-023-02916-5

**Published:** 2023-10-31

**Authors:** Katie J. Neale, Hannah M. O. Reid, Barbara Sousa, Erin McDonagh, Jamie Morrison, Sandy Shultz, Eric Eyolfson, Brian R. Christie

**Affiliations:** 1 Division of Medical Sciences, University of Victoria, Medical Sciences Building,3800 Finnerty Road, Victoria, BC V8P 5C2 Canada; 2 Vancouver Island University, 900 Fifth Street, Nanaimo, BC V9R 5S5 Canada; 3 Monash Trauma Group, Monash University, Melbourne, Australia; 4 Institute for Aging and Life Long Health, University of Victoria, 3800 Finnerty Road, Victoria, BC V8P 5C2 Canada; 5 Island Medical Program, Cellular and Physiological Sciences, University of British Columbia, 3800 Finnerty Road, Victoria, BC V8P 5C2 Canada; 6 Djavad Mowafaghian Centre for Brain Health, University of British Columbia, 3800 Finnerty Road, Victoria, BC V8P 5C2 Canada

**Keywords:** Concussion, Dentate gyrus, Awake closed-head injury, Microglia, Sub-granular zone, Development, mTBI, Mild traumatic brain injury

## Abstract

Childhood represents a period of significant growth and maturation for the brain, and is also associated with a heightened risk for mild traumatic brain injuries (mTBI). There is also concern that repeated-mTBI (r-mTBI) may have a long-term impact on developmental trajectories. Using an awake closed head injury (ACHI) model, that uses rapid head acceleration to induce a mTBI, we investigated the acute effects of repeated-mTBI (r-mTBI) on neurological function and cellular proliferation in juvenile male and female Long-Evans rats. We found that r-mTBI did not lead to cumulative neurological deficits with the model. R-mTBI animals exhibited an increase in BrdU + (bromodeoxyuridine positive) cells in the dentate gyrus (DG), and that this increase was more robust in male animals. This increase was not sustained, and cell proliferation returning to normal by PID3. A greater increase in BrdU + cells was observed in the dorsal DG in both male and female r-mTBI animals at PID1. Using Ki-67 expression as an endogenous marker of cellular proliferation, a robust proliferative response following r-mTBI was observed in male animals at PID1 that persisted until PID3, and was not constrained to the DG alone. Triple labeling experiments (Iba1+, GFAP+, Brdu+) revealed that a high proportion of these proliferating cells were microglia/macrophages, indicating there was a heightened inflammatory response. Overall, these findings suggest that rapid head acceleration with the ACHI model produces an mTBI, but that the acute neurological deficits do not increase in severity with repeated administration. R-mTBI transiently increases cellular proliferation in the hippocampus, particularly in male animals, and the pattern of cell proliferation suggests that this represents a neuroinflammatory response that is focused around the mid-brain rather than peripheral cortical regions. These results add to growing literature indicating sex differences in proliferative and inflammatory responses between females and males. Targeting proliferation as a therapeutic avenue may help reduce the short term impact of r-mTBI, but there may be sex-specific considerations.

## Introduction

Traumatic brain injuries (TBI) are a significant health issue worldwide, and the vast majority of these injuries (~ 80%) are classified as a mild (mTBI). These injuries commonly occur following accidents, in collision sports, military duty, and following cases of domestic violence [1, 2]. Known as silent injuries, mTBIs produce transient behavioral deficits lasting 1–2 weeks, but do not produce skull fractures or brain bleeds [3]. The age at injury can influence recovery timelines, with children and adolescents requiring longer periods to recover [4, 5]. As juveniles have the highest rates of mTBIs, it is important to understand the mechanisms of injury in this population [6]. This is particularly true since repeated-mTBIs (r-mTBI) early in life may underlie more serious degenerative disorders later in life, including dementias, Alzheimer’s disease, and Parkinson’s disease [7–9]. Currently sex differences in the underlying pathophysiology of mTBI are poorly understood, in part due to the majority of preclinical models focusing on adult males [10, 11], and the use of animal models that tend to produce more severe injuries than normally occur with mTBI [12].

Sex differences in response to mTBI have been documented in both clinical and preclinical studies [13–16], and evidence suggests that females experience more injuries than males in sex comparable sports [13]. The neural correlates of sex differences in cognitive and affective domains are not well understood, but maturational and functional differences in the brains of males and females may contribute to this heterogeneity. Females tend to present with longer symptom duration and more neurocognitive and emotional symptoms [17–20]. Males on the other hand, present have more motor deficits and cognitive disorientation, performing worse on executive functioning tasks. Male performance is also worse with a prior history of concussion [21], so manipulating timelines of inter-injury intervals in repeated injury paradigms can be critical for teasing apart these heterogeneous outcomes. Indeed, significant sex differences from multiple injuries have been observed whether they occur in a single day [22–24], 24 h apart [25, 26], 48 h apart [27, 28], 72 h apart [29, 30], or 96 h apart [31].

One brain region linked to the heterogeneity of outcomes following r-mTBI is the hippocampus. The hippocampus is a critical brain structure that is involved in spatial navigation, learning and memory processes, and affective behavior [32, 33], and has been shown to be particularly sensitive to the diffuse effects of mTBI [28, 34]. Selective hippocampal neuronal loss, changes in hippocampal excitability, and impaired hippocampal synaptic plasticity have been observed in a variety of models of experimental TBI [35]. We have previously shown that closed-head injuries can impair spatial memory for at least one week after injury in juvenile rats [24], the duration of any behavioral deficits can dependent on the severity of the injury model and the age at injury.

An emerging trend in hippocampal research views hippocampal functioning along the dorsal and ventral axis as being involved in different types of behavior. For example, the dorsal hippocampus seems to more associated with spatial learning [36] while the ventral hippocampus seems to play a greater role emotional regulated behaviors [37]. The hippocampus is also one of the few brain regions that undergoes neurogenesis into adulthood, and this process has also been found to play a role in different behaviors [38, 39]. These functional differences, and the different behaviors associated with post-concussive syndrome, suggest that dorsal and ventral dentate gyrus segments could have different proliferative responses to r-mTBI [28, 34].

Traumatic brain injuries induce time-, sex-, and age-dependent neuroinflammatory cascades [25, 26], and these neuroinflammatory cascades are mediated by infiltrating immune cells, astrocytes, and microglia [40–45]. Ionized calcium binding adaptor molecule 1 (Iba1) is commonly used as a marker of microglia and macrophages and, depending on the model used, Iba1-positive cells have shown to increase, decrease, or not change following injury (For review see [46]). With repeated injuries, it is believed that microglia are “primed” by the initial injury, and this can then exacerbate the neuroinflammatory response to subsequent injuries and be associated with worse functional outcomes [47]. That is, when microglia are repeatedly activated, their response can become pathological and impair spatial learning [48], as well as the process of neurogenesis [49]. Under homeostatic conditions microglia can regulate neurogenesis by phagocytosing neural progenitor cells (NPC) [50]. While there is evidence that TBI can increase NPC proliferation, these cells may not survive to become functionally integrated [51–53]. Interestingly, if microglia are activated by TBI, then they can phagocytose both NPCs and the progeny of NPCs [50].

While several studies in a variety of preclinical models have found enhanced cell proliferation after TBI [54, 55], there remains a paucity of data on any sex differences in hippocampal cell proliferation following TBI. This may be important, as the inflammatory response of microglia and astrocytes can be more robust in males than in females after TBI [25, 56, 57]. Sexual dimorphism has been observed in both glial cell number and morphology, as well as in the pro-inflammatory cytokine expression after TBI [56]. Furthermore, the time course of the inflammatory response is different in males and females [56, 58, 59]. Generally, studies show that males have a more rapid response than females, which occurs in a region-specific manner [56, 57].

This study investigated the proliferative response in juveniles to r-mTBI using an awake closed-head injury (ACHI) model. The ACHI avoids the use of anesthetic agents that have been shown to have neuroprotective properties [60, 61], allowing us to examine the effects of r-mTBI on the juvenile rat brain during a time period when it undergoes critical neurodevelopmental and organizational processes that are mediated, in part, by microglia and astrocytes. It is unclear if the young brain is more or less susceptible to secondary damage after insult, and what the long-term outcomes are [62–64]. Given that sex differences in the pathophysiological response and functional recovery following mTBI are observed in clinical and preclinical populations [65], this study directly compares the response in females and males. Future treatment and therapies, including those that look at neuroprotective mechanisms, will benefit from a greater understanding of how females and males differ in their response to rmTBI. Thus, this work seeks to determine whether there are sex differences in the acute proliferative response following r-mTBI.

## Methods

### Animals

Approval for all animal procedures was obtained from the University of Victoria Animal Care Committee and complied with Canadian Council on Animal Care (CCAC) standards. A total of 56 Long-Evans rats were used in this study (28 females, 28 males). Dams with litters of 10–12 pups at postnatal day (PND) 13–15 were purchased from Charles River Laboratories (St. Constant, PQ). The animals were acclimatized to the animal care center at the University of Victoria for one week before weaning at PND21. The unit used standard cage housing at 22.5 °C ± 2.5 °C, with ad libitum access to food and water, kept on a 12-h light/dark cycle. Animals were randomly assigned to either sham or r-mTBI groups and killed on 1 or 3 days post-injury (14 males and 14 females in each group, resulting in 7 animals per time point) and housed with 2–3 sex- and condition-matched littermates. All procedures occurred between 7:30 am and 11:30 pm.

### Awake closed-head injury and neurologic assessment protocol

Following weaning on PND21 animals were habituated to restraint cones for one week before the day of testing. The cones had an opening near the nostril to allow for ventilation and the restraint cone was closed near the rear of the animal using a plastic hair clip. A restraint score was tabulated for each procedure to record for the ease of animal entry into the bag. On PND28 animals were randomly assigned to receive eight ACHI or sham injuries over 16 h (once every 2 h). Previous work in our lab has demonstrated injury-induced deficits in neurological assessment protocol (NAP) following administration of eight injuries over 24, 48, and 96 h [66]. The goal of the current study was to standardize post-injury days given that previous cell proliferation studies result in transient changes [66, 67]. The ACHI procedure was performed as previously described [68, 69]. In brief, animals were immobilized using a clear plastic restraint cone. A flat rubber tip was attached to the end of an impactor arm (modified Leica Impact One controlled cortical impactor, Leica Biosystems Inc., ON, Canada) and targeted a 3D-printed helmet that was placed over the left parietal cortex. This helmet distributes the force of the acceleration evenly across the skull, producing a diffuse injury. The modified impactor was activated, rapidly depressing the head into a foam pad at a velocity of 6.0 m/s (injury depth 10 mm, dwell time 10 ms). Animals were immediately removed from the restraint cone and a pain score was recorded after each NAP procedure. Pain scores were recorded from 0, displaying no signs of pain, to a maximum score of 6; any obvious signs of pain (abnormalities in: locomotion, social behavior, breathing, palpation of the impact site and skin turgor) would result in animal removal. No animals were removed in the current study. Sham injuries were performed under the same conditions, however, the impactor arm was activated next to the animals head. Following the last procedure, animals were returned to their home cage and monitored for either 1 or 3 days post-injury.

On PND27 and following each ACHI or sham procedure, animals were assessed using a neurological assessment protocol (NAP). As previously described [68] the NAP evaluated the level of consciousness, basic reflexes, and sensorimotor function. The NAP consists of 3 measures for loss of consciousness (tail pinch reflex, apnea, righting reflex; in seconds) and four sensorimotor tasks (startle response, limb extension, beam walk, and rotating beam). As the NAP was performed immediately after each impact, the investigator was not blinded to injury group. All tasks were measured one after the other and took a couple minutes. We scored the sensorimotor functions on a four-point Likert scale from 0 (fail) to 3 (perfect), resulting in a possible total score of 12.

### BrdU injections

On either PID1, or PID3, a single dose of the thymidine analogue bromodeoxyuridine (BrdU; 200 mg/kg), was administered 2 h prior to animals being killed. We, and others, have previously shown this is a saturating dose that effectively labels the available progenitor pool in the hippocampus [38, 70]. BrdU was dissolved in 0.1 M phosphate-buffered saline (PBS) at 20 mg/ml. The injection occurred approximately 12 h after the final injury on PID1, or 60 h later on PID3.

### Perfusion and brain extraction

All animals were administered an overdose of isoflurane (> 5%, inhalant) 2 h following BrdU administration, and once unresponsive to a toe pinch, they were perfused transcardial with 0.1 M PBS followed by 4% paraformaldehyde (PFA) using a gravity feed perfusion system. Brains were post-fixed in PFA for 24 h before being transferred to a cryoprotectant solution (30% sucrose) until saturated (defined as having sunk to the bottom of the solution after several days). Saturated brains were then stored in 0.1 M PBS and 0.01% sodium azide until used for histology.

### Histology and immunohistochemistry

Brains were sectioned at 50 µm and stored as 1 in 6 series in a six well-plate in 0.1 M PBS and 0.01% sodium azide. We maintained the dorsal–ventral order of each series for analyses. Using coordinates obtained from the Paxinos and Watson Rat Brain Atlas [71], the dorsal hippocampus was defined as − 2.28 mm to − 4.74 mm from bregma. The ventral hippocampus was defined as − 4.74 mm to − 6.48 mm from bregma, as described previously [72, 73].

### Cresyl violet staining

Cresyl violet acetate (ThermoFisher, J64318-09) is a basic aniline dye that interacts with the nucleic acid content of cells that is highly concentrated in the endoplasmic reticulum and ribosomes of cells, as well as the DNA content of the cell nucleus, in healthy cells. Staining is reduced, and structural loss is usually clearly evident, when there is damage or neurodegeneration [22, 74, 75]. One of the 1 in 6 series of sections was rinsed in a series of descending ethanol concentrations (100% 5 min, 95% 1 min, 70% 1 min) to reduce membrane lipids, prior to being immersed in 0.5% cresyl violet acetate solution (pH 6). A distilled water rinse was then performed to remove excess dye, and a final rinse in 1% acetic acid solution was performed to clear any residual dye not absorbed by cells. Finally, sections were dehydrated using a progressive series of ethanol concentrations; cleared using xylene; and then cover-slipped using Permount (Fisher Chemical, SP15-100).

### Immunohistochemistry

Primary antibodies against BrdU (MAB3424), and the endogenous proliferation indicator, Ki-67 (Ab15580), were optimized prior to being used for these experiments (see Table 1), and a separate 1 in 6 series of tissue sections was used for each primary antibody. BrdU staining: endogenous peroxidase activity was quenched in slices by immersing them in 0.6% H2O2 for 30 min. Next, the DNA was denatured to allow the primary antibody to access BrdU sites by incubating sections in 10 mM sodium citrate buffer and 50% formamide for 1 h at 65 °C. Sections were then immersed in a 10 mM sodium citrate buffer followed by 2 N hydrochloric acid (HCl; 37 °C; 1 h) to break protein cross-links. To neutralize the HCl, slices were transferred into 0.1 M borate buffer and then rinsed in 0.1 M TBS three times. To block non-specific antibody binding, sections were incubated in 3% Normal Goat Serum (in 0.1 M TBS and 0.25% Triton X-100) for 1 h at room temperature, and then transferred to an identical solution that also contained the primary antibodies. At this step, the well-plates were placed on a Belly Dancer to provide gentle agitation and incubated at 4 °C overnight. Following washes in blocking buffer and TBS, we immersed sections in a biotinylated secondary antibody (Ab1893) for 2 h. We then placed sections in an avidin–biotin-peroxidase complex (Vectastain ABC Elite) to allow immunopositive cells to be visualized using DAB (3,3′-diaminobenzidine tetrahydrochloride; Vector laboratories Burlingame, CA). Ki-67 staining: Ki-67 staining was performed using identical steps to those above; however, the antigen retrieval was done in 10 mM sodium citrate at 85 °C for 30 min, and a quenching step was done on day 2 in 3% H_2_O_2_ for 15 min. The primary antibody used for Ki-67 was (Ab15580; see Table 1).

**Table 1:** Experimental antibodies

Primary antibody	Company	CAT#	RRID	Concentration used (%Serum)	Time in primary (h)	Secondary (source)
Mouse anti-BrdU (DAB)	Millipore Sigma	MAB3424	AB_94851	1:4000 (3%)	16	Goat anti Mouse (Millipore Sigma B6649)
Rabbit anti-Ki67 (DAB)	Abcam	Ab15580	AB_443209	1:1000 (5%)	48	Goat anti Rabbit (Millipore Sigma B8895
Sheep anti-BrdU (IF)	Abcam	Ab1893	AB_302659	1:1000	16	Goat anti Sheep Biotin (Invitrogen A24565)
						Streptavidin Alexa Fluor 488 conjugate (ThermoFisher S11223)
Rabbit anti-Iba1 (IF)	Wako	019-19741	AB_839504	1:1000	16	Donkey anti Rabbit, Alexa Fluor 568 (ThermoFisher A10042)
Mouse anti-GFAP (IF)	Millipore Sigma	G3893	AB_477010	1:1000	16	Donkey anti Mouse Alexa Fluor 647 (Millipore Sigma, AP192SA6)

### Immunofluorescence triple staining

We stained a subset of sections for BrdU, Iba1 (ionized calcium-binding adaptor molecule), and glial fibrillary acidic protein (GFAP) to allow for BrdU positive cells to be phenotyped. Iba1 is expressed by macrophages and microglia, while GFAP is a type III intermediate filament protein expressed by astrocytes. This provides a means to quantify the proportion of Brdu + cells that also stain positive for the microglia/macrophage and astrocyte antibodies. DNA denaturation followed the same protocol as DAB staining; however, because we used HRP conjugated antibodies, PBS was used instead of TBS. Following denaturation, sections were blocked in 3% bovine serum albumin (BSA) with 0.25% Triton X-100 in PBS for 1 h at room temperature. They were then incubated overnight in the primary antibodies (see Table 1) at 4 °C. Following washes in PBS and blocking solution, sections were incubated in the biotinylated goat anti-sheep antibody for 2 h, then in fluorophore secondary antibodies for 2 h at room temperature. Finally, sections were rinsed and mounted in 60% 2,2′-thiodiethanol (TDE), which has a high refractive index, making it advantageous for high-resolution confocal microscopy.

### Profile counting in the subgranular zone (SGZ)

BrdU + and Ki-67 + cells were quantified using profile counting on an Olympus brightfield CX21 microscope (Olympus Corporation, Center Valley, PA, USA) and a 100X oil immersion lens. Counts were restricted to cells in the SGZ (within 2–3 cell diameters of the granule cell layer; approximately 50 µm). The researcher was blinded to group identity, PID and sex. Because Ki-67 + and BrdU + cells tend to be distributed heterogeneously and often in clusters, high magnification (100x) was required to distinguish individual cell bodies that overlapped. The average number of cells from all slices was calculated and multiplied by the number of dorsal or ventral DG sections (48 sections to represent total cells per dorsal DG, 33.6 sections to represent total cells per ventral DG). Thus, cell counts are the mean BrdU + or Ki-67 + cells per entire dorsal or ventral DG ± SEM. Representative images were acquired with an Olympus brightfield BX51 microscope (MBF Bioscience, Williston, VT, USA) using StereoInvestigator software version 11.03 (MBF Bioscience, Williston, VT, USA).

### Assessment of BrdU staining in the hippocampus

A custom macro in FIJI-Image J (Version 1.52p, National Institutes of Health, USA) was used to threshold images and count BrdU particles. The macro was applied to images taken on an Olympus brightfield BX51TF microscope with a 10X objective. Images of whole tissue sections were compiled using StereoInvestigator software version 11.03 (MBF Bioscience, Williston, VT, USA) for *n* = 5 animals/group. The hippocampal sections were classified as dorsal or ventral sections based on bregma coordinates. The contrast of each image was optimized, and the background was subtracted to identify cell-specific maxima (pixel values of 255). Speckle-patterns of particles unified for individual cells by running the fill holes command before the analyze particles command for a specific size (0–60 pixels^2^). Data are presented as mean BrdU + cells per mm^2^ ± SEM.

### Cell type characterization

To determine whether the BrdU + cells were glial cells, sections from *n* = 3 animals/group were triple stained and analyzed for co-localization of BrdU with either Iba1 or GFAP. Two slices in the series were selected, and the entire DG was imaged using an Olympus BX61WI confocal laser scanning microscope and Olympus FluoView FV10-ASW 1.7c software (Olympus Corporation, Center Valley, PA, USA) with a 20X objective at 1.5X zoom (NA = 0.75, 1024 × 1024, 0.41 µm/pixel), 1.0 µm *z* steps and Kalman filtering (mean = 2). Because of the low number of BrdU + cells in sham animals, all BrdU + cells were identified in each stack, then characterized as BrdU + /Iba1-/GFAP-, BrdU + /Iba1 + /GFAP- or BrdU+/Iba1−/GFAP+. Data are presented as % Cell Type of Total BrdU + cells ± SEM.

### Statistical analysis

All statistical analysis was conducted using R (R Core Team, Vienne, Austria, 2019) or GraphPad Prism version 8 (GraphPad Software, San Diego, CA, USA). Graphs were generated using Graphpad Prism 8. Appropriate tests (Levene’s test for homogeneity of variance; Shapiro–Wilk or QQ plots for normality) were applied in each condition to ensure assumptions were not violated, and decisions to use parametric or non-parametric tests were determined as appropriate.

## Results

### Acute neurological deficits are present following each ACHI, but do not increase in severity with repeated administration

**Fig. 1:**
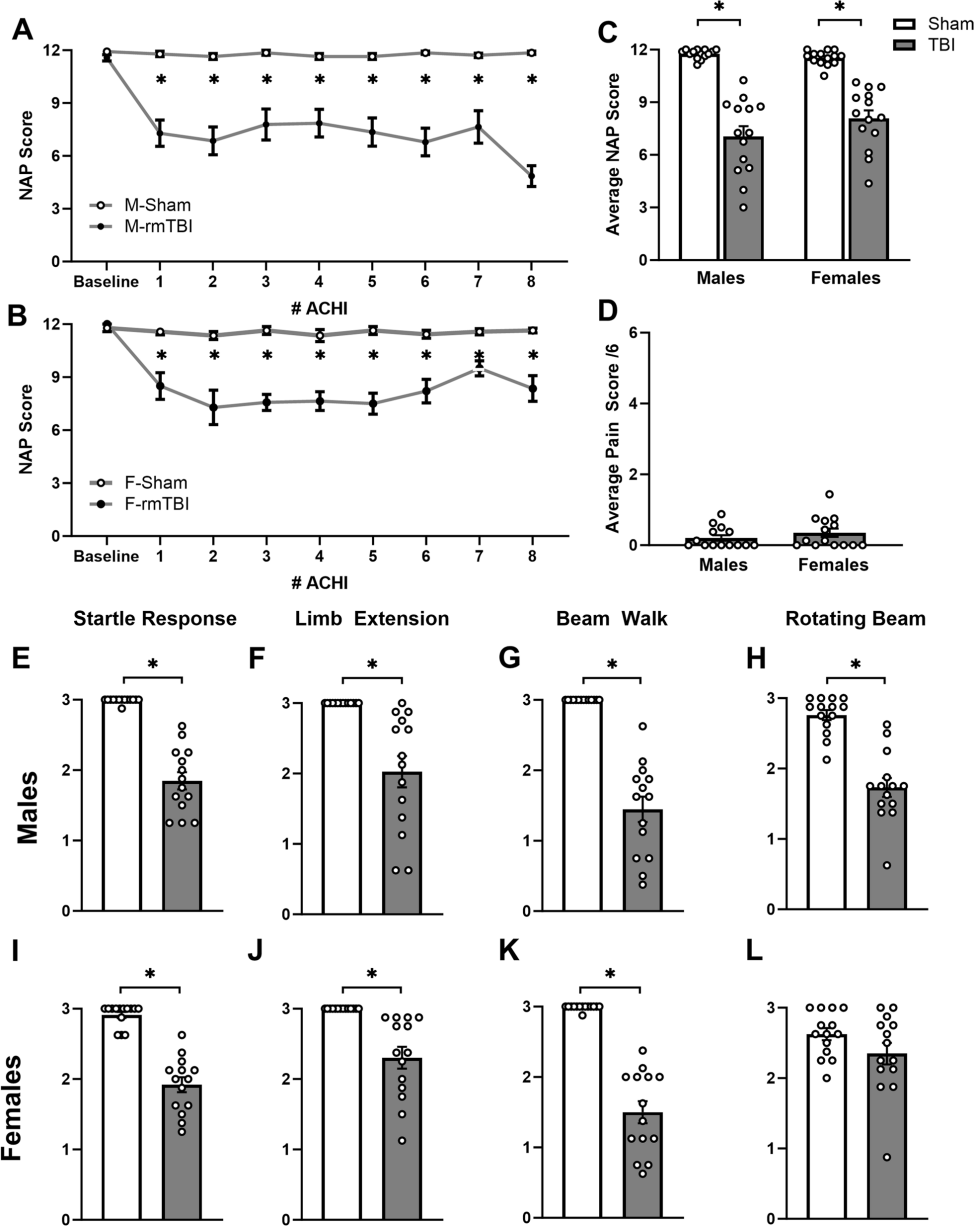
r-mTBI induced by ACHI causes acute neurological impairment. Male (**A**) and female (**B**) animals were administered an ACHI, followed by a NAP, eight times on PND28. The r-mTBI animals had significantly lower NAP scores across all eight ACHIs that did not increase significantly with each additional ACHI. **C** The average NAP scores for males and females following TBI did not differ significantly from each other, but were significantly lower than for Sham animals for both sexes. **D** Prior to each ACHI, animals were assessed for pain and abnormal behavior. Scores of 0–1 reflect no abnormalities up to a maximum score of 12; males and females showed low average pain scores. No animal scored higher than 1.5, indicating there were no significant indicators of pain in these cohorts. **E–L** The 4 reflexive and sensorimotor tasks were scored on a scale of 0 (fail) to 3 (perfect score). Males (**E–H**) and females (**I–L**) showed significant impairments on startle response, limb extension and beam walk. Only males showed impairment on the rotating beam task. Group data are presented as mean ± SEM. Open circles in bar graphs represent individual animal data points

**Fig. 2:**
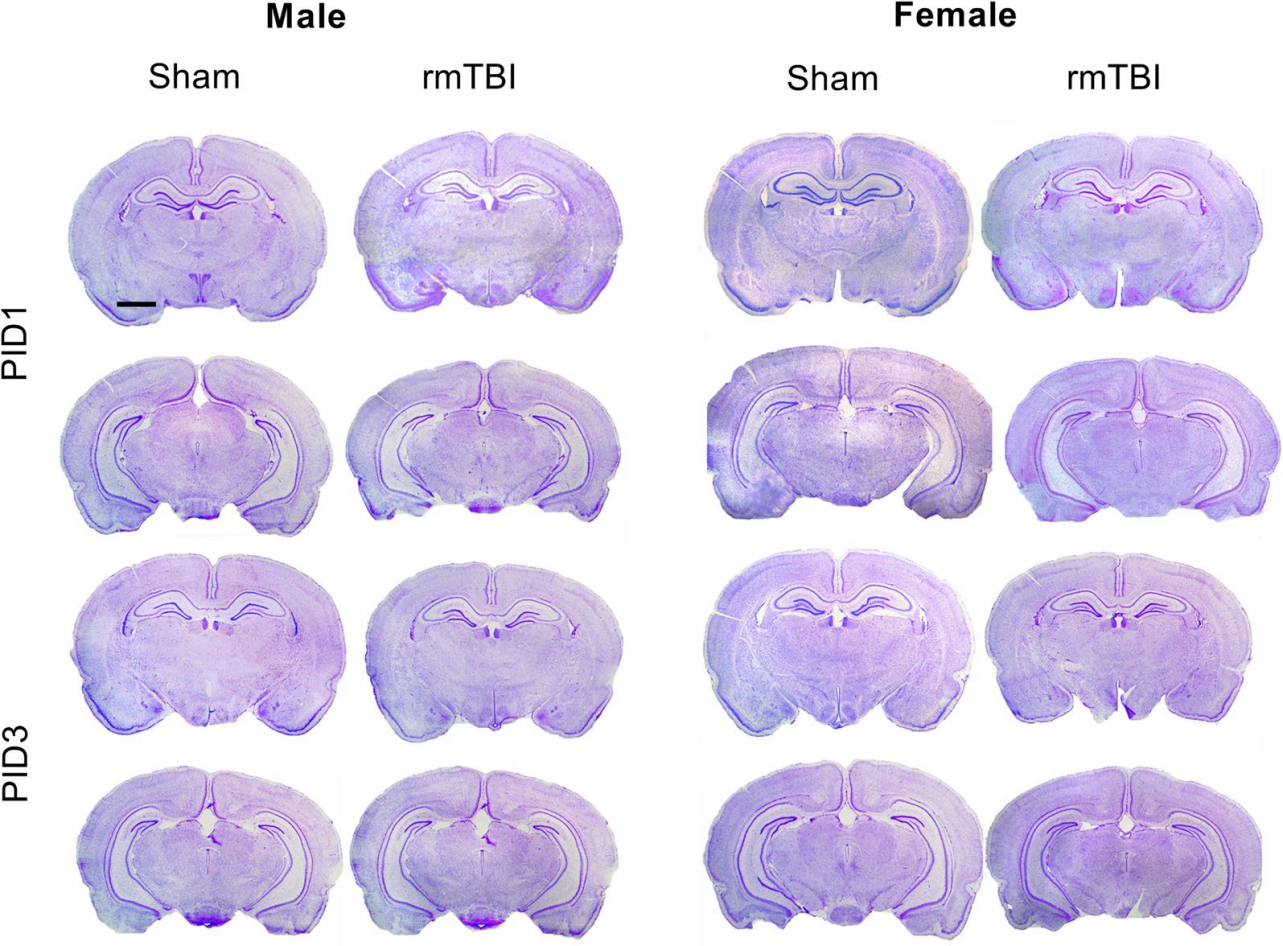
Repeated mTBI using the ACHI model does not produce significant structural damage. Cresyl violet stain of representative coronal sections following administration of the ACHI procedure (8×) are shown. Photomicrographs display both the dorsal (upper) and ventral (lower) hippocampus for male and female animals at PID1 and PID3. In neither sex were signs of cortical contusion or major tissue damage apparent. Scale bar (upper left): 2 mm

All animals were administered either an ACHI, or exposed to the sham procedure, eight times over a 16-h period. All subjects were assessed for a series of four sensorimotor and reflex tasks (startle response, limb extension, beam walk and rotating beam walk), administered immediately following each ACHI/sham procedure as a neurological assessment procedure (NAP) [68, 69]. There were no sex differences or differences between groups at baseline (Fig. 1A, B), however, after each ACHI, both male and female r-mTBI animals showed significant impairment on their NAP scores (two-way repeated measures ANOVA with Geisser–Greenhouse correction for sphericity: *F*_1,26_ = 66.17, *p* < 0.001 [males], *F*_1,26_ = 51.99, *p* < 0.001 [females]). Averaged total NAP scores across all eight ACHIs indicated that male and female r-mTBI animals were significantly impaired compared to shams (Fig. 1C). With the exception of female performance on the rotating beam task (*p* = 0.092), both male and female animals showed impaired performance on all four sensory motor tests (Mann–Whitney test; *p* < 0.001; Fig. 1E–H [males], I–L [females]). There was no indication of cumulative damage with r-mTBI, as the magnitude of the deficits did not increase significantly across the eight ACHI procedures. In addition, as shown in Fig. 2, no overt signs of tissue damage were apparent in cresyl violet-stained sections from either female or male animals following r-mTBI.

### *Repeated mTBI produces a transient increase in BrdU* + *cells in the subgranular zone*

**Fig. 3:**
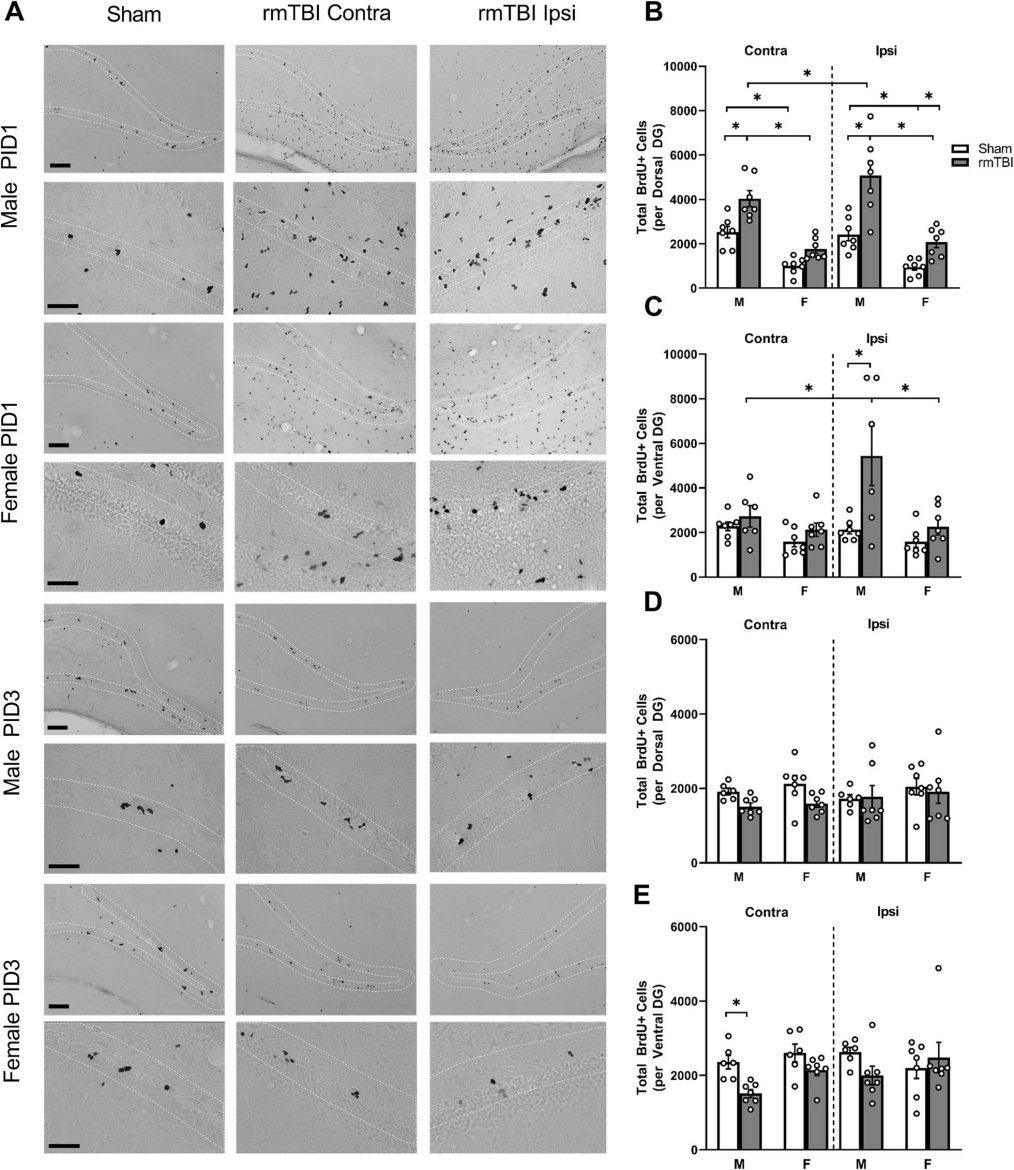
Regional and sex-specific transient increases in BrdU + cells following r-mTBI.** A** Representative images of the dorsal SGZ (white dotted line) for Sham and r-mTBI animals at PID1 and PID3 in males and females. Images taken of SGZ’s contra (middle column) and ipsi (right column) to the impact hemisphere showing BrdU + cells. Note the bilateral proliferative response to r-mTBI. **B** At PID1, there was an increase in BrdU + cells in the contra and ipsi-SGZ of male r-mTBI animals compared to Shams. Male r-mTBI animals showed a significantly higher number of BrdU + cells compared to female r-mTBI animals in contralateral and ipsilateral SGZ. In the ventral SGZ at PID1 (**C**) there was a significantly greater number of BrdU + cells in male r-mTBI animals in the ipsilateral hemisphere. There was a significant increase in BrdU + cells in the dorsal ipsilateral SGZ of female r-mTBI animals. **D**, **E** By PID3, there was no longer a significant difference in the number of BrdU + cells in either the dorsal or ventral SGZ of male r-mTBI animals. Images were taken at 10× (upper panels) and 40X (lower panels); scale bars are 50 µm and 25 µm, respectively. Data presented as mean ± SEM. Open circles in bar graphs represent individual animal data points

To investigate the effects of r-mTBI on cellular proliferation in the neurogenic niche of the hippocampus, we performed a profile count of BrdU + cells in the SGZ (Fig. 3A). Cell counts were performed to allow separate quantification of the dorsal dentate gyrus (dDG; Bregma: − 2.28 to − 4.74 mm) and ventral dentate gyrus (vDG; Bregma: − 4.74 to − 6.48 mm) at PID1 in the contralateral and ipsilateral hemispheres. A three-way ANOVA indicated a main effect of group, with r-mTBI animals exhibiting an increase in BrdU + cells in both the dDG (*F*_1,24_ = 28.08, *p* < 0.0001) and vDG (*F*_1,23_ = 11.28, *p* = 0.003; Fig. 3B, C). There was also a main effect of sex, with increased proliferation observed specifically in the dDG and vDG of male animals (*F*_1,24_ = 52.45, *p* < 0.0001; *F*_1,23_ = 11.45, *p* = 0.003). In both the dorsal and ventral DG, there was an interaction between group and hemisphere (*F*_1,24_ = 6.374, *p* = 0.019; *F*_1,23_ = 5.036, *p* = 0.035). Male animals showed a significant increase in BrdU + cells in the ipsilateral dDG (*p* < 0.0001) and vDG (*p* < 0.0001), while female animals showed a significant increase in the ipsilateral dDG only (*p* = 0.017). Male animals also showed a significant increase in the contralateral dDG (*p* = 0.002), but this was not observed in female animals. No significant increase in the number of BrdU + cells was observed in the contralateral vDG of either male or female r-mTBI animals. Finally, in the vDG there was a significant three-way interaction (*F*_1,23_ = 4.228, *p* = 0.051) indicating a combined effect between group, side and sex. Overall, these results indicate a more robust proliferative response, particularly in the ipsilateral DG, of male r-mTBI animals and a reduced response in female r-mTBI animals at PID1.

To determine whether this increase in BrdU + cells in the SGZ of male r-mTBI is maintained throughout the acute phase after injury, BrdU + cells were also counted in animals injected with BrdU on PID3. In these animals, BrdU + cell numbers were not significantly different from those observed in sham animals in both the dDG and the vDG. A three-way mixed ANOVA revealed no main effects for BrdU + cells in r-mTBI animals in the dDG. A significant interaction between group and side was found (*F*_1,23_ = 4.591, *p* = 0.043), but no comparisons of interest were found (Fig. 3D). In the vDG, there was a main effect of group (*F*_1,23_ = 4.749, *p* = 0.040), and post hoc analysis revealed a decrease in BrdU + cells in the contralateral DG of male r-mTBI animals compared to shams (Fig. 3E). These results indicate that the robust increase in BrdU + cells observed at PID1 following r-mTBI does not persist through to PID3.

### *An increase in Ki-67* + *cells confirms a robust proliferative response in males at PID 1*

**Fig. 4:**
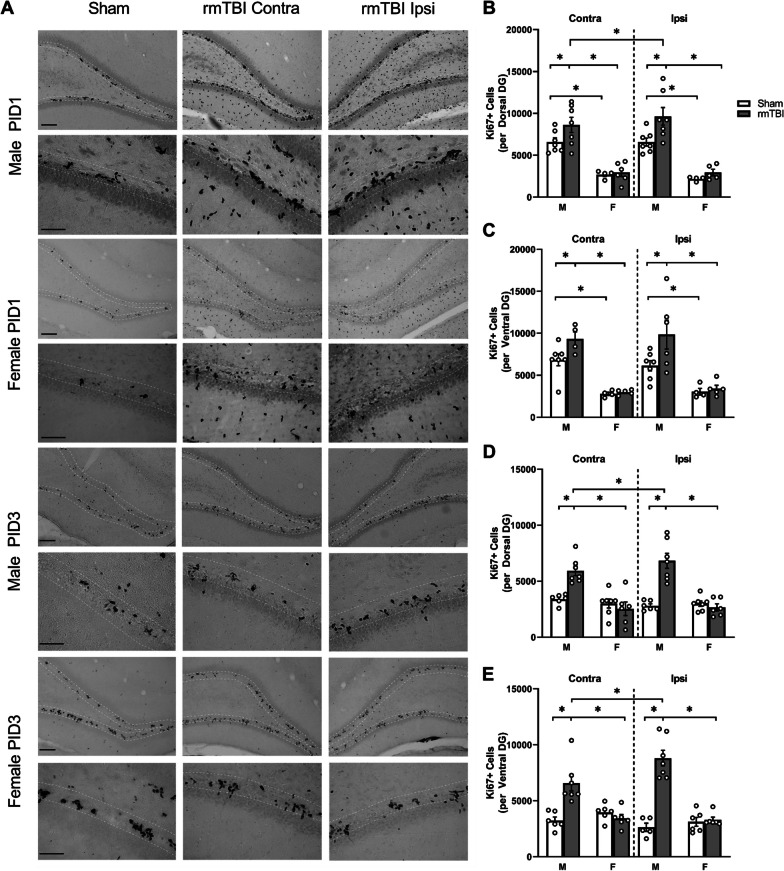
Persistent increase in Ki-67 + cells in the dorsal and ventral SGZ of males following r-mTBI.** A** Representative images of the dorsal SGZ (white dotted line) for Sham and r-mTBI animals at PID1 and PID3 in males and females. Contra (middle column) and ipsi (right column) SGZs with Ki-67 + ve cells are shown. **B, C** At PID1, r-mTBI males showed an increase in Ki-67 + cells in the dorsal DG (**B**) and ventral DG (**C**). This increase was seen in the contra- and ipsi-SGZ for r-mTBI males, but not in either hemisphere in r-mTBI females. D, E This increase in Ki-67 + cells was also observed at PID3 in the dorsal and ventral SGZ of r-mTBI males. Females did not show any significant difference in Ki-67 + cell numbers at PID3. Images were taken at 10 × and 40 ×; scale bars are 50 µm and 25 µm, respectively. Data presented as mean ± SEM. Open circles in bar graphs represent individual animal data points

The BrdU injection paradigm used in this study captures a very specific (approx. 2 h) window of cell division [38]. To investigate levels of cell proliferation without a specific temporal paradigm, an antibody against Ki-67, an endogenous marker of cell proliferation was used (Fig. 4A). At PID1, a three-way ANOVA revealed main effects of both group (*F*_1,20_ = 4.419, *p* = 0.048) and sex (*F*_1,20_ = 57.61, *p* < 0.0001), and an interaction between side and sex (*F*_1,19_ = 6.351, *p* = 0.0208) on Ki-67 + cells in the dorsal DG (Fig. 4B). Male r-mTBI animals showed an increase in Ki-67 + cells in the ipsilateral (*p* = 0.002) and contralateral (*p* = 0.03) SGZ compared to shams as well as side-matched female r-mTBI animals (*p* < 0.0001 [ipsi]; *p* < 0.0001 [contra]). The response in the ipsilateral dDG was greater than in the contralateral dDG (*p* = 0.005). At this PID1 time point, female r-mTBI animals did not show an increase in Ki-67 + cells in either hemisphere. Interestingly, there were sex differences in sham levels of Ki-67 + cells, as male sham animals showed significantly higher numbers of proliferating cells compared to female shams in both hemispheres (*p* = 0.0002 [ipsi]; *p* = 0.0008 [contra]). As with the dDG, the ventral DG revealed main effects of group (*F*_1,18_ = 5.001, *p* = 0.038) and sex (*F*_1,18_ = 34.42, *p* < 0.0001) and an interaction between side and sex (*F*_1,14_ = 5.457, *p* = 0.035) were observed (Fig. 4C). Post hoc analysis revealed a significantly higher number of Ki-67 cells in the contralateral (*p* = 0.004) and ipsilateral (*p* = 0.005) vDG of male r-mTBI animals compared to shams. This increase in Ki-67 + cells was not observed in female r-mTBI animals. As in the dorsal DG, male sham animals and r-mTBI animals had significantly greater numbers of Ki-67 + cells compared to females (*p* = 0.0346 [ipsi sham], *p* = 0.0054 [contra sham], *p* < 0.0001 [ipsi r-mTBI and contra r-mTBI]).

### *Increase in Ki-67* + *cells in males persists at PID 3*

The number of Ki-67 + cells was counted at PID3 to determine whether the number of cells in the active stage of the cell cycle would be increased over a prolonged period. The increase in male r-mTBI animals at PID1 appeared to persist until PID3 in both hemispheres of the dorsal and ventral DGs (Fig. 4D, E). Indeed, a three-way ANOVA revealed main effects of group (*F*_1,22_ = 13.69, *p* = 0.001 [dDG]; *F*_1,21_ = 33.31, *p* < 0.0001 [vDG]) and sex (*F*_1,22_ = 25.19, *p* = 0.001 [dDG]; main effect sex *F*_1,21_ = 21.83, *p* = 0.0001 [vDG]) as well as interactions between group and sex (*F*_1,22_ = 22.50, *p* < 0.0001 [dDG]; *F*_1,21_ = 33.80, *p* < 0.0001 [vDG]) and group and side (*F*_1,22_ = 4.935, *p* = 0.037 [dDG]; *F*_1,20_ = 10.18, *p* = 0.005 [vDG]). There was also an interaction between sex and side, but in the ventral DG only (*F*_1,20_ = 5.379, *p* = 0.031). Post hoc analysis confirmed greater numbers of Ki-67 + cells in male r-mTBI animals in the ipsilateral and contralateral DGs compared to shams (*p* < 0.0001 [ipsi dDG]; *p* < 0.0001 [ipsi vDG]; *p* = 0.0001 [contra dDG]; *p* < 0.0001[contra vDG]) and compared to female r-mTBI animals (*p* < 0.0001 [ipsi dDG]; *p* < 0.001 [ipsi vDG]; *p* < 0.0001 [contra dDG]; *p* < 0.0001 [ipsi vDG]). Additionally, this response in male r-mTBI animals was significantly greater in the ipsilateral DG compared to the contralateral DG (*p* = 0.018 [dorsal], *p* = 0.0004 [ventral]). Overall, this indicates a prolonged and more robust proliferative response in male r-mTBI animals. Notably, by PID3 (which corresponds to PND31), there were no longer sex differences in the number of Ki-67 + cells in sham animals.

### Cell proliferation is not restricted to the DG at PID 1

**Fig. 5:**
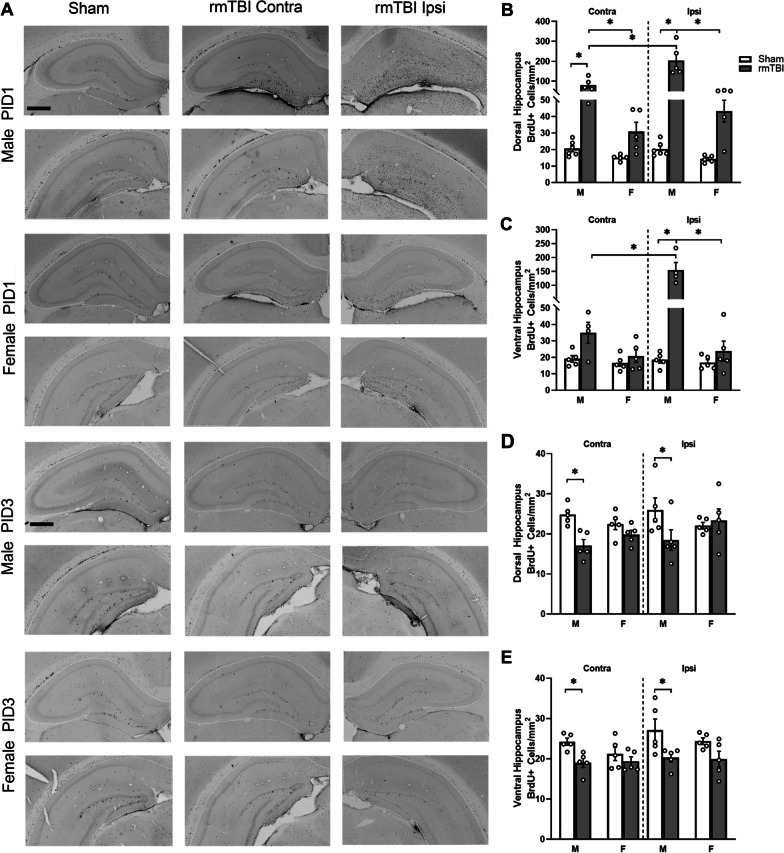
Cell proliferation is not restricted to the SGZ at PID1. **A** Representative images of BrdU staining for the dorsal and (top in each pair) and ventral (bottom) hippocampus of Sham and r-mTBI animals at PID1 and PID3 in males and females. Contra (middle column) and ipsi (right column) are shown side by side to highlight the bilateral response that is especially robust in the ipsi hemisphere. **B, C** Quantification of this response by BrdU + cells in the hippocampus showed increased numbers of BrdU + cells in the dorsal hippocampus of r-mTBI males. This increase was significantly greater than Shams in the contra and ipsi hippocampus, though the increase in BrdU + cells in the ipsi hippocampus was significantly greater than the contra hippocampus of r-mTBI males. The ventral hippocampus did not show quite as large an increase and the response to r-mTBI was significantly higher in the ipsi hippocampus only. R-mTBI females showed a non-significant increase. **D, E** By PID3, there was a significant decrease in BrdU + cells in r-mTBI males compared to Shams. This was observed in the dorsal and ventral hippocampus. Representative images were taken at 10X, scale bar is 500 µm. Data presented as mean ± SEM. Open circles in bar graphs represent individual animal data points

The initial aim of this study was to investigate changes in cellular proliferation in the neurogenic niche after r-mTBI. However, extensive proliferation was observed throughout most sections with both BrdU and Ki-67 at PID1 that centered around the mid-brain, as opposed to the outer cortical regions. While the response extended well beyond the structure of the hippocampus, the proliferative response was the most robust in the DG which was the focus of our analysis. To quantify the proliferative response, 10 × images were taken from sections stained with BrdU, the brain region was carefully traced, and then a particle analysis protocol was applied in imageJ (Fig. 5A). A three-way ANOVA revealed main effects of group (*F*_1,17_ = 37.54, *p* < 0.0001 [dDG]; *F*_1,16_ = 7.296, *p* = 0.016 [vDG]), sex (*F*_1,17_ = 22.21, *p* = 0.0002 [dDG]; *F*_1,16_ = 5.765, *p* = 0.029 [vDG]) and side (*F*_1,17_ = 46.83, *p* < 0.0001 [dDG]; *F*_1,16_ = 10.28, *p* = 0.005 [vDG]) as well as a three-way interaction between factors (*F*_1,17_ = 31.46, *p* < 0.0001 [dDG]; *F*_1,16_ = 9.278, *p* = 0.008 [vDG]) in both the dorsal and ventral hippocampus at PID1 (Fig. 5B, C). All two-way interactions were significant in both the dorsal and ventral hippocampus. In both hemispheres of the dorsal hippocampus, there was a significant increase in BrdU + cells for male r-mTBI animals compared to sham males (*p* < 0.0001 [ipsi]; *p* = 0.002 [contra]) and r-mTBI females (*p* < 0.0001 [ipsi]; *p* = 0.011 [contra]). There was a non-significant increase in BrdU + cells for female r-mTBI animals, though representative images point to females also exhibiting a small proliferative response outside the SGZ (Fig. 5A, rows 3–4). For male r-mTBI animals, the number of BrdU + cells in the ipsilateral hippocampus was significantly higher compared to the contralateral hippocampus, similar to what was observed with BrdU and Ki-67 profile counts at PID1 (*p* < 0.0001). Thus, although there are bilateral increases in proliferation in the hippocampus, this is significantly greater in the side ipsilateral to impact site. Indeed, post hoc tests in the ventral hippocampus revealed a robust ipsilateral response: male r-mTBI animals showed increased BrdU + cells in the ipsilateral hippocampus compared to shams (*p* < 0.0001), r-mTBI females (*p* < 0.0001), and the contralateral hippocampus of r-mTBI males (*p* < 0.0001).

By PID3, a decrease in the number of BrdU + cells in the male r-mTBI animals compared to shams was observed according to post hoc tests for a three-way ANOVA that revealed main effects of group in the dorsal (*F*_1,16_ = 16.42, *p* = 0.0009) and ventral (*F*_1,16_ = 12.68, *p* = 0.003) hippocampus (Fig. 5D, E). This decrease was apparent in both ipsilateral (*p* = 0.011 [dDG]; *p* = 0.004 [vDG]) and contralateral (*p* = 0.009 [dDG]; *p* = 0.023[vDG]) hemispheres.

### Triple labeling with GFAP and Iba1 reveals a transient inflammatory response at PID1 in r-mTBI males and females

The SGZ of the DG is a major site of cell proliferation and neurogenesis in the adult brain, and the majority of these cells co-label with early neuronal markers or immature neuron markers [70, 76, 77]. Other types of proliferating cells in the DG include activated radial glial cells, progenitor cells (i.e., cells that do not yet express early neuronal or immature neuronal markers), microglia, and oligodendrocytes. Few, if any astrocytes are normally found dividing [70]. With a disruption to the brain, like r-mTBI, the increased number of dividing cells may be comprised more heavily of glial cells. Alternatively, progenitor cells may experience an accelerated cell cycle, or more progenitors may be actively proliferating [78]. To investigate the cell phenotype of these dividing cells in male r-mTBI animals at PID1, a triple stain analysis with BrdU, Iba1 and GFAP was performed to identify dividing microglia and macrophages and/or dividing astrocytes or radial glial cells.

**Fig. 6:**
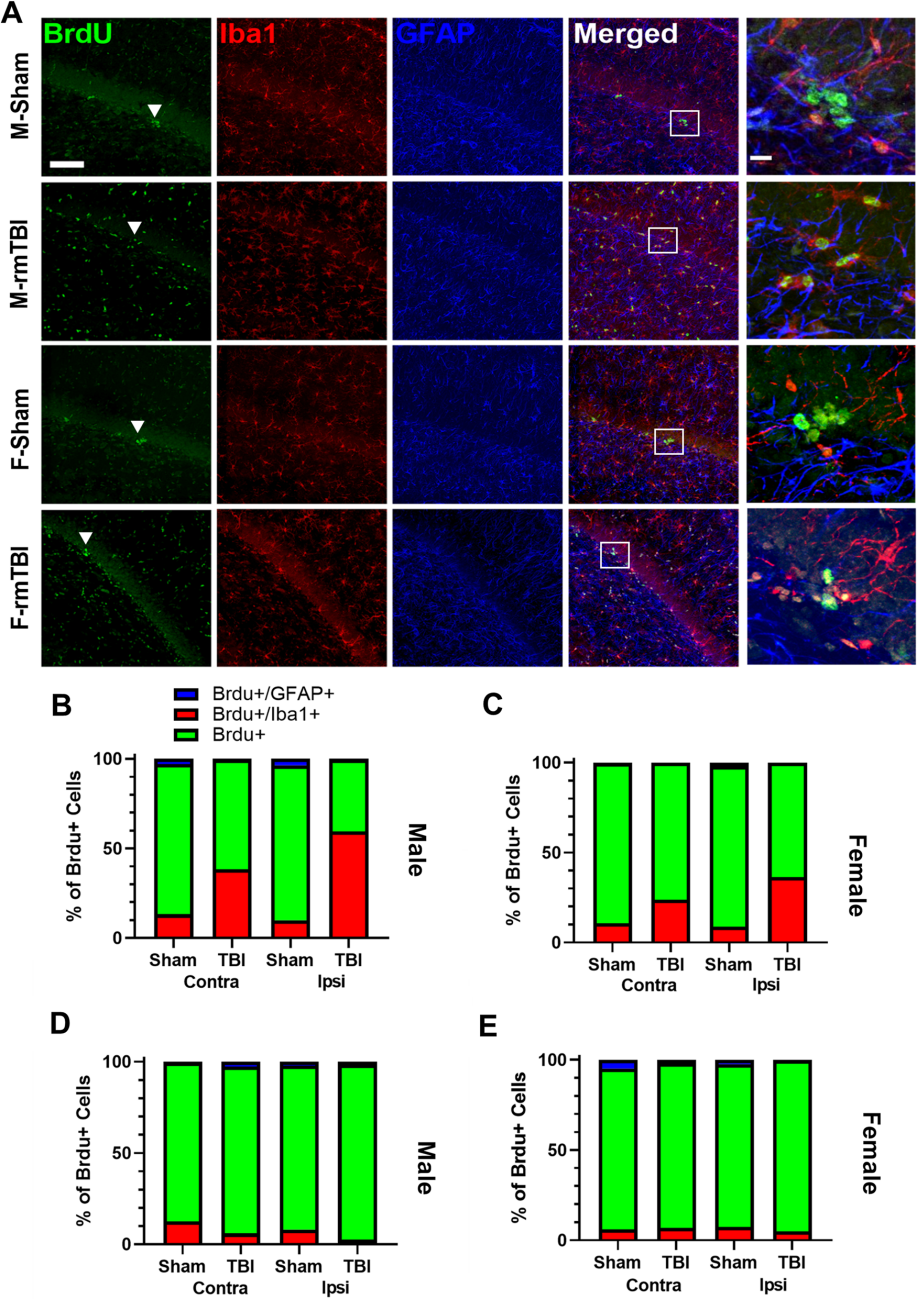
At PID1 a greater proportion of BrdU + cells were co-labeled with Iba1 + in r-mTBI males and females compared to Shams. **A** Representative images of triple labeling with BrdU (green, left column), Iba1 (red, second column), GFAP (blue, third column) and merged (fourth column) shown for the ipsilateral SGZ. Right column (expanded view of white rectangle from merged) shows representative BrdU + cells that were identified (white triangles in BrdU column) and examined for co-localization with Iba1 and/or GFAP. Scale bar is 10 µm. **B, C** A PID1, there was a greater proportion of BrdU + /Iba1 + /GFAP- cells in r-mTBI males and females compared to Shams. This indicates that this is likely an inflammatory response rather than exacerbated neurogenic proliferation. **E, F** At PID3, most BrdU + cells identified as BrdU + /Iba1-/GFAP- in r-mTBI animals, and there was no longer any difference in BrdU + cell phenotypes between r-mTBI and Sham animals. Scale bar is 10 µm. For triple labeling quantification, 1 dorsal and 1 ventral slice (approx. Bregma − 3.36 mm and Bregma − 5.28 mm, respectively) were chosen for imaging from *n* = 3 animals per group. Dorsal and ventral slices were averaged and presented as an n of 1; data are represented as mean ± SEM and individual points represent 1 animal. All BrdU + cells were identified in the DG from 20× stacks obtained using confocal microscopy. Scale bar: 100 µm

An exhaustive sampling approach was used due to the rarity of BrdU + cells in sham animals (Table 2). BrdU + cells were then characterized as BrdU + /Iba1-/GFAP- (not a dividing glial cell), BrdU + /Iba1 + /GFAP− (dividing microglia or macrophage) or BrdU + /Iba1 −/GFAP + (dividing astrocyte or radial glial cell). Very few BrdU + /Iba1 −/GFAP + cells were identified, however many cells at PID 1 were BrdU + /Iba1 + /GFAP − indicating an inflammatory profile rather than neurogenic proliferative response (Fig. 6A). A three-way mixed effects ANOVA with group, sex and side as factors revealed significant main effects of group (*F*_1,8_ = 152.4, *p* < 0.0001), sex (*F*_1,8_ = 19.35, *p* = 0.0023), and side (*F*_1,8_ = 14.64, *p* = 0.005), on percentage of BrdU + /Iba1 + cells in the SGZ relative to total BrdU + cells at PID1 (Fig. 6B, C). There were also significant interactions between group and sex (*F*_1,8_ = 13.16, *p* = 0.0067), and group and side (*F*_1,8_ = 27.32, *p* = 0.0008), on the percentage of BrdU + /Iba1 + cells in the SGZ. Post hoc analysis revealed a significantly higher percentage of BrdU + /Iba1 + cells in the SGZ of male r-mTBI animals in both contralateral and ipsilateral hemispheres compared to shams (both *p* < 0.0001). Additionally, the ipsilateral SGZ of male r-mTBI animals had a significantly higher percentage of BrdU + /Iba1 + cells as compared to the contralateral SGZ (*p* = 0.0005). Interestingly, the contralateral and ipsilateral SGZ in female r-mTBI animals had a significantly higher percentage of BrdU + /Iba1 + cells as compared to shams (*p* = 0.0069 and *p* < 0.0001, respectively) despite a non-significant increase in BrdU + cells as shown with the DAB staining experiments. The proportion of BrdU + /Iba1 + cells was found to have returned to sham levels by PID3 (Fig. 6D, E). No significant main effects or significant interactions were observed; in male and female animals, the majority of cells were BrdU + /Iba1-/GFAP-.

**Table 2:** Results of immunohistochemical staining

Sex	Stain	Sham		TBI	
		Contralateral count (%)	Ipsilateral count (%)	Contralateral count (%)	Ipsilateral count (%)
PID1					
Male	Brdu +	45.67 (*83.72*)	42.67 (*86.37*)	54.33 (*61.11*)	57.50 (*40.13*)
	Iba1 +	8.17 (*13.31*)	5.00 (*9.94*)	38.00 (*38.29*)	83.83 (*59.37*)
	Gfap +	1.50 (*2.97*)	1.83 (*3.69*)	0.67 (*.60*)	0.67 (*.50*)
Female	Brdu +	25.17 (*88.91*)	32.33 (*89.16*)	36.83 (76.18)	30.17 (*63.55*)
	Iba1 +	3.33 (*10.75*)	3.50 (*8.91*)	11.33 (*23.82*)	17.50 (*26.45*)
	Gfap +	0.17 *(.34*)	0.83 (*1.93*)	0.00 (*0*)	0.00 (*0*)
PID3					
Male	Brdu +	31.67 (*87.04*)	28.50 (89.86)	27.33 (*91.39*)	33.33 (*96.01*)
	Iba1 +	4.17 (*12.44*)	2.50 (8.09)	1.83 (*5.96*)	1.00 (*2.54*)
	Gfap +	0.17 (*.52*)	0.50 (2.04)	1.00 (*2.66*)	0.50 (*1.45*)
Female	Brdu +	34.00 (*89.17*)	25.00 (*90.19*)	26.33 (*91.14*)	41.50 (*94.79*)
	Iba1 +	1.33 (*5.98*)	1.83 (*7.40*)	2.00 (*6.85*)	2.50 (*4.97*)
	Gfap +	1.50 (*4.85*)	0.50 (*2.41*)	0.50 (*2.01*)	0.17 (*0.24*)

Non-italicised numbers are actual cell counts; italicised numbers reflect percentages

## Discussion

Despite evidence of sex differences following mTBI in clinical populations, females remain underrepresented in preclinical research [10]. Males have been the primary focus of preclinical research, perhaps due to their higher involvement in collision sports. Despite this, emerging evidence indicates that females may experience more mTBIs than males in sex comparable sports [15, 79, 80], and reveal the presence of sex differences in symptomology presentation [15, 79, 81], symptomology duration [19, 20], and symptomology onset [82]. Among the most common symptomologies following mTBI are those involved in the cognitive and affective domains that require hippocampal functioning [83]. The hippocampus is highly susceptible to the diffuse sheering and tearing forces associated with mTBI [84, 85], and contains the dentate gyrus, one of the few brain regions to display persistent neurogenesis through adulthood [86]. As the hippocampus is a heterogeneous structure with regions along the dorsal/ventral axis being responsible for different behavioral domains, we sought to determine if there were also regional differences in the proliferative response to mTBI. The purpose of this study was to investigate heterogeneity of neurogenesis and inflammatory response in the SGZ and dentate gyrus from female and male juvenile rats following r-mTBI. The current study used gold standard markers of cellular proliferation with BrdU and Ki-67. In this study we show time-, sex- and region-dependent alterations in proliferative response to juvenile r-mTBI. In brief, enhanced cellular proliferation was observed in males following r-mTBI. R-mTBI enhanced proliferation more in the dorsal than in the ventral hippocampus. We also show that males displayed an increased inflammatory response following injury, as compared to females, and that the majority of Brdu + cells were co-labeled as Iba1 + . These results indicate that in the acute period following injury, females and males do not display similar proliferative and inflammatory responses. As cellular proliferation has been an area of interest for therapeutics, these findings suggest the need for tailored sex-specific therapies.

Use of NAP scoring is a viable tool for assessing post-concussive symptomologies immediately following an mTBI. While we did not see exacerbation of scores in both females and males across the eight injuries, those subjected to r-mTBI had significantly lower scores than sham counterparts. A previous study in our lab compared single mTBI to r-mTBI and observed poorer NAP performance in those animals subjected to r-mTBI [75]. A subsequent study utilizing a different injury paradigm (two injuries a day for 4 days) also did not see worsening NAP scores after each subsequent injury [24]. Following any individual mTBI, there exists in a period of cerebral vulnerability where subsequent injuries can exacerbate outcomes [87]. This includes, but not limited to, microglial priming and an enhanced pro-inflammatory response [88]. At this time, it is not clear how well NAP scores correlate with other behavioral deficits or pathophysiological responses. It is possible that the use of more subtle behavioral tests, such as gait analysis, may be more sensitive to detect worsening deficits across impacts.

In the dentate gyrus, environmental experiences like voluntary exercise and environmental enrichment can enhance cellular proliferation [33, 38]. The juvenile period in particular is associated with significant growth and maturation and therefore, the rate of neuronal proliferation is enhanced compared to adolescents and adults [89]. However, a requirement for enhanced plasticity during this developmental period may also leave the juvenile brain more susceptible to the long-term negative effects of a mild brain injury. Our data, and that of others, indicates that the effect of injury appears to be time- and region-dependent [62, 90, 91]. The amount of cellular proliferation depends in part on the model utilized, the time point examined following injury, and method used to measure cell proliferation. In this study we found that males who sustained r-mTBI displayed increased expression of BrdU + and Ki-67 + cells in the SGZ at PID1 and PID3. R-mTBI-induced increases in cellular proliferation in males are congruent with previous research. Nueberger and colleagues administered moderate TBI in juvenile males and found increased Ki-67 + cells in the DG on PID3 [92]. Enhanced proliferation following injury appears to be similar in studies utilizing adult and late adolescent animals and may extent up to 31 days post-injury [67, 93, 94]. Additionally, our results expand on the findings by Clark and colleagues who saw increased Ki-67 + cells in the ipsilateral hemisphere following lateral fluid percussion mTBI by showing an increase in Ki-67 + cells in the contralateral hemisphere, lending to the diffuse nature of the ACHI [67]. Interestingly, the pattern of cell proliferation overall suggested that the proliferation was associated with a movement around the center of mass of the brain. This is in contrast to the “coup-contre-coup” model of brain injury that is commonly described, as there was no evidence for enhanced proliferation under the “impact” site or on the opposite of the brain.

Interestingly, when measuring the expression of BrdU + cells in the entire dentate gyrus we saw an inverse effect between PID1 and PID3. Following r-mTBI, expression of BrdU + cells was increased at PID1 but decreased at PID3. This is different to the expression of Ki-67 + cells which displayed increased expression at both time points. These discrepancies between expression of BrdU + and Ki-67 + cells may be attributed to the exogenous and endogenous nature of these markers, respectively. Ki-67 is a marker of the entire cell cycle while BrdU is a marker of the S-phase of the cell cycle [95, 96]. These diverging results between expression of BrdU + and Ki-67 + cells have also been demonstrated following pediatric mTBI. Diaz-Chavez and colleagues administered CCI at P21 and noticed no increase in expression of Ki-67 + cells, but a decrease in expression of BrdU + cells at PID21 in the subgranular cell layer [97]. However, a critical difference in study design, in addition to the age of the animals used, was the timing of BrdU injection. The current study injected BrdU 2 h prior to killing while Diaz-Chavez and colleagues injected BrdU 10 days prior to killing and thus would not quantify only the initial proliferative response.

To our knowledge, data showing r-mTBI-induced changes in neuronal proliferation in females are sparse. Our study revealed that females did not experience the robust increases in cellular proliferation makers observed in males following r-mTBI. These findings are similar to Yamakawa and colleagues who found no change in Ki-67 + cells in females in the GCL at PID17 [29]. Thus, the reduced response in females appears to span juvenile and adolescent ages and models of injury (ACHI and lateral impact model). Sex differences in cellular proliferation seen in this study could be explained by the proximity to puberty. The average age of onset of puberty for females and males is P35 and P45, respectively [98]. While this would be at the early end of the spectrum, adolescence is associated with decreases in cellular proliferation. Siddiqui and Romero investigated sex differences in cellular proliferation in juveniles (P30), adolescents (P45), and adults (P70). The researchers found increased Ki-67 + cells in males at P30 before equalizing at P45 and P70 [99]. Juvenile sex differences are also seen in neonatal males and females [100, 101]. The current study corroborates the Siddiqui and Romero findings at P30; independent of injury, males displayed enhanced cellular proliferation compared to females. Interestingly this effect was region-dependent in dorsal slices. This effect could be due to hippocampal outputs to the prefrontal cortex and amygdala which undergo significant maturation in adolescence [102, 103].

While we were able to demonstrate increased mitotic activity up to PID3 following injury, we were unable to determine the survival of those cells at chronic time points. Enhanced cell proliferation up to PID3 is significant as it may reflect an innate compensatory mechanism that could help the brain recover from minor injuries. Previous reports using lateral fluid percussion mTBI indicate a greater number of BrdU + cells ipsilateral (but not contralateral) in the SGZ up to PID31 [94]. This indicates that the proliferating cells we see in the DG are not dying soon after, and could play an important role in recovery. Although more studies will need to examine the influence of these surviving cells on behavior. Changes in cellular proliferation are commonly seen following stressors. The hippocampus has a high concentration of glucocorticoid receptors, which leaves the hippocampus highly susceptible to stress [104]. Both acute and chronic stressors influence adult neurogenesis in the dorsal dentate gyrus, whereby, males present with reduced expression of BrdU + cells following both acute predator odor stress and chronic restraint stress, while females present with no changes [105, 106]. Although it appears that the timing of stressors is important, as adolescent chronic restraint stress shows reductions in expression of BrdU + cells in adulthood for females but enhanced expression of BrdU + cells in males [107]. While not directly investigating the influence of r-mTBI, we can assume the induction of mTBI to be an acute stressor. Following experimental fluid percussion mTBI in adult males, expression of plasma corticosterone have been shown to both decrease [108] and increase [109] on PID1. In these experiments, we have to acknowledge the possibility that restraint bag habituation could act as a chronic stressor, resulting in enhanced HPA axis activation. Restraint stress is often used in both acute and chronic stress paradigms, but those studies administer much longer restraint protocols (20 min [1];1 h [2]; 3 h [3]; 6 h [4]) than our study (< 1 min a day) [110–112]. Chronic stress has been associated with decreased cell proliferation in the dentate gyrus [113–115], whereas in the current study r-mTBI resulted in enhanced cell proliferation. Therefore, it is likely the changes in cell proliferation were due to the effects of r-mTBI given the significant differences in stress timings and enhanced, not decreased cellular proliferation.

Inflammation is a characteristic of all severities of TBI. While an initial pro-inflammatory response is critical for recovery, issues arise when a pro-inflammatory response is left unabated (for review see [116]). We utilized immunofluorescence to characterize BrdU + cells with markers often involved in an inflammatory response, Iba1 to label microglia and macrophages, and GFAP to label astrocytes. Our triple staining results revealed the majority of cells were BrdU + /Iba1 + at PID1 in both females and males following r-mTBI. This inflammatory response was greater in males and in the ipsilateral hemisphere. Interestingly, by PID3 this increase in co-labeled BrdU + /Iba1 + cells returned to sham levels in both females and males. This is similar to what is seen within the field, where studies display a transient increase reactive glial cells labeled with BrdU [94, 117]. There is a twofold way of thinking about our triple staining results. First, it is possible that following r-mTBI neural progenitor cells differentiated into microglia. Secondly, it is possible that microglia phagocytosed neural progenitor cells following r-mTBI. Recently microglial nomenclature has come under scrutiny suggesting that Iba1 + microglia are not necessarily activated [118]. We now know these are highly dynamic cells that express a number of transcription factors. Only utilizing Iba1 limits our abilities to determine if the microglia in the SGZ are phagocytic or not. However, if microglia are reducing the pool of neural stem cells/progenitor cells future neurogenesis may be impaired. Based on the timepoints chosen for this experiment, we are not able to determine what occurs at more chronic timepoints. Dependent on the inflammatory profile (anti or pro) neurogenesis can be reduced or enhanced, respectively. While our triple labeling experiment provides information as to whether the majority of these cells are glial in nature, it cannot provide further information about neuronal fate or neuronal subtype. Further triple stain analysis with progenitor cell or immature neuron markers such as Sox2 or DCX would enable a more definitive conclusion as to the subtype of BrdU + /Iba1−/GFAP− cells.

## Conclusions

The ACHI model used to induce r-mTBI in juvenile rats produced an injury that is consistent with the clinical definition of sports related concussion. There was transient LOC, and short-lived impairment of neurological function in the absence of major structural damage. This demonstrated that r-mTBI results in acute neurological impairment in both sexes without hippocampal structural damage. Assessment of cell proliferation showed that r-mTBI resulted in robust and widespread cellular proliferation in males. This response was greater in males than in females and persisted until PID3. While cells localized to the SGZ are generally progenitor cells, characterization of cell types in both males and females showed a heightened inflammatory response at PID1. The sex differences in the acute proliferative and inflammatory response to r-mTBI reported here provide novel information about the response of the juvenile hippocampus following r-mTBI. Future studies will help to inform whether these sex differences contribute to changes in functional outcomes in neurogenesis and learning and memory. Understanding what is neuroprotective and what is harmful regarding the heightened hippocampal response in males after r-mTBI and the minimal response in females will provide exciting opportunities for intervention. These novel findings highlight a need for the inclusion of females in preclinical and clinical TBI research. Future studies will continue to inform how military personnel exposed to blast waves, the many women affected by intimate partner violence, and athletes engaged in contact sport may better recover from r-mTBI.

### Transparency, rigor, and reproducibility statement

 Preregistration was not performed as it was not possible to do in a meaningful way for this exploratory preclinical pathophysiological study. A total of 56 Long-Evans rats were purchased from Charles River Breeders for this study and randomly assigned to control and experimental groups. No animals died as a result of treatment in this study. Microscopy analyses were performed by K.N., H.R., B.S., and E.M. with the individuals blinded as to the group condition of the tissue sample. Analyses were performed by K.N. after blinding for subject identity was removed. Experiments were conducted using a modified CCI device and the modifications are described elsewhere [22, 69]. All other reagents are available commercially, and detailed staining protocols are available upon request. Histological samples from these animals are no longer available, but additional samples can be easily generated for other research groups upon request. The authors agree to provide the full content of the manuscript upon request by contacting the corresponding author.

## Data Availability

Data are available upon request to the corresponding author.
